# The Causes and Treatment of Infantile Diarrhea

**Published:** 1894-07

**Authors:** 


### Selections and Abstracts.

---

The Causes and Treatment of Infantile Diarrhea.—The *Journal de Clinique et de Therapeutique Infantiles* for May 24th publishes a paper on this subject by Dr. G. Variot, in which he says that the majority of infants who are attacked with diarrhea and vomiting are those artificially fed, or those who are given solid food at a time when their digestive organs have not attained their full activity and power. Where there is hereditary syphilis or scrofulo-tuberculosis, the children are more susceptible to digestive troubles, as those affections greatly impair the young organism, the digestive organs of which are in a constantly changing condition. In these cases diarrhea is only an epiphenomenon, and it is not astonishing if it resists the usual means for combating it, for it may be combined with tuberculous lesions of the intestine or of the mesenteric ganglia.

Children nursed exclusively at the breast are rarely attacked with persistent diarrhea, although, even in this case, too frequent nursing will cause a gastro-intestinal dyspepsia, accompanied by repeated vomiting and diarrhea. A constant change also of wet nurses is, again, a cause of this affection in infants.

With regard to solid food, physicians have insisted that milk is the only diet for infants, but it seems almost impossible, says Dr. Variot, to impress this simple fact, particularly upon the common people, who, in France, and in America, according to Dr. L. Emmet Holt, obstinately cling to the idea that the sooner the children are fed on solid food the quicker they will grow. It is generally admitted that food should not be given to infants before dentition occurs. At this age the salivary and intestinal glands are powerless to saccharize the starchy substances, which irritate the mucous membranes and undergo injurious fermentations. In some cases which had come under the author's observation, where solid food had been given to the children, the abdomen was distended and dilated, and at the linea alba there was a true eventration, so that one or two fingers could be introduced between the two recti muscles, and this eventration indicated that the abdominal wall had been in some way forced open by the flatulent distention of the intestines. This flatulence was without doubt connected with the abnormal gaseous fermentation of the starchy substances.

Another cause of diarrhea in infants is the quality of the milk, which is often inferior, and the milk which is sold to the poor, especially in large cities, is often dirty and adulterated. Much sickness and many deaths also are attributed to the use of the nursing bottles with long rubber tubes, which are so difficult to clean properly, and to the rapid alterations of the milk, into which the breath of the child is constantly passing; the milk also is often diluted with impure water and contains septic germs which provoke very dangerous fermentation. The statistics of death from diarrhea, drawn up by different observers, all go to show that the mortality is greater in June, July, and August, and at this time the digestive organs should be carefully watched, particular attention being paid to the quality of the milk and to its sterilization; if necessary, the quantity should be somewhat reduced at each meal. Some writers have observed epidemics of diarrhea, and some cases of contagion. Although not much importance is attached to the latter, it is well, the author says, to take children to the country during hot weather. Another cause of diarrhea is dentition, the effects of which should not be neglected. The author does not share the popular idea that diarrhea from this cause is salutary and needs no attention; at this time the digestive organs of infants should be carefully watched. As soon as the diarrhea appears the child should not nurse oftener than every three hours; if it is artificially fed the milk should be diluted with a third of lime-water. Before the child nurses a tablespoonful of the following mixture should be given: Distilled water, two ounces; lactic acid, half a dram; tannin dissolved in alcohol, eight grains; and syrup of quince, five drams.

For a child fed artificially, the author always prescribes sterilized milk, and gives careful directions for the antiseptic cleansing of the bottle. The best proof, he says, that diarrhea, if it is treated from the first day of its appearance, is due to the bad quality of the milk, is that it yields to treatment in two or three days after the administration of sterilized milk. In some of the author's cases this was the only agent used to effect a recovery from attacks of diarrhea due to bad milk, toast-water, etc. If sterilized milk is properly prepared and of good quality, it is the best food for children artificially fed, as the sterilization destroys all morbid or noxious germs, and the author has observed that, as a rule, it is well borne by infants.—*N. Y. Med. Journal.*

